# Percutaneous Aspiration of a Mobile Infected Thrombus from the Right Ventricular Outflow Tract Using the AngioVac System

**DOI:** 10.1155/2019/6279019

**Published:** 2019-04-17

**Authors:** Justin Ugwu, Umbreen Hussein, Sampson Alliu, Ravi Gurujal

**Affiliations:** ^1^Department of Hospital Medicine, Miami Valley Hospital, Dayton, Ohio, USA; ^2^Cardiovascular Disease Fellowship, Wright State University, Dayton, Ohio, USA; ^3^Department of Cardiovascular Medicine, Maimonides Medical Center, Brooklyn, New York, USA; ^4^Department of Cardiology, Miami Valley Hospital, Dayton, Ohio, USA

## Abstract

The AngioVac system was invented in 2012 and was originally designed for the removal of thrombi from the venous system. It has been successfully used in the management of iliocaval and right endocardial thrombi but is reportedly less effective in the management of pulmonary emboli (PE). Since its advent, there has been interest in its application towards other medical situations. One of the most revolutionary uses thus far has been for percutaneous debridement of valvular and cardiac electronic device-associated vegetations. In most instances, the AngioVac device has been used to obviate the need for surgery in high-risk patients. Here, we describe a novel use of this device in the successful retrieval of a large, mobile, infected thrombus from the right ventricular outflow tract in a high surgical-risk patient.

## 1. Introduction

An isolated right ventricular (RV) outflow mass is a rare finding in clinical practice. The differential diagnosis of a mass appearing six days after a negative transesophageal echocardiogram (TEE) in this location includes thrombus, embolus, tumor embolus, and endocardial vegetation. Frequently, initial management options are conservative, including anticoagulation for thrombus/embolus and antibiotics for vegetations. When surgical intervention is indicated, thrombectomy, embolectomy, or vegetectomy is usually invasive and associated with increased morbidity and mortality. As described in several case reports, the AngioVac catheter has proven to be a suitable alternative to surgery in these scenarios [1–3].

## 2. Case Description

**Figure 1:**
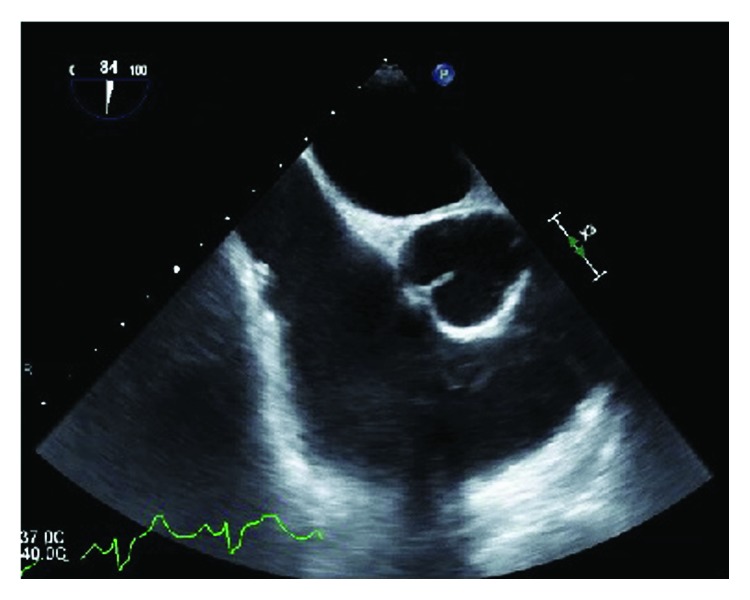
Midesophageal short axis view obtained on transesophageal echocardiogram performed on hospital day 3 with a focus on the right atrium, tricuspid valve, and RVOT. There was no clear, identifiable source of infection on this initial evaluation.

**Figure 2:**
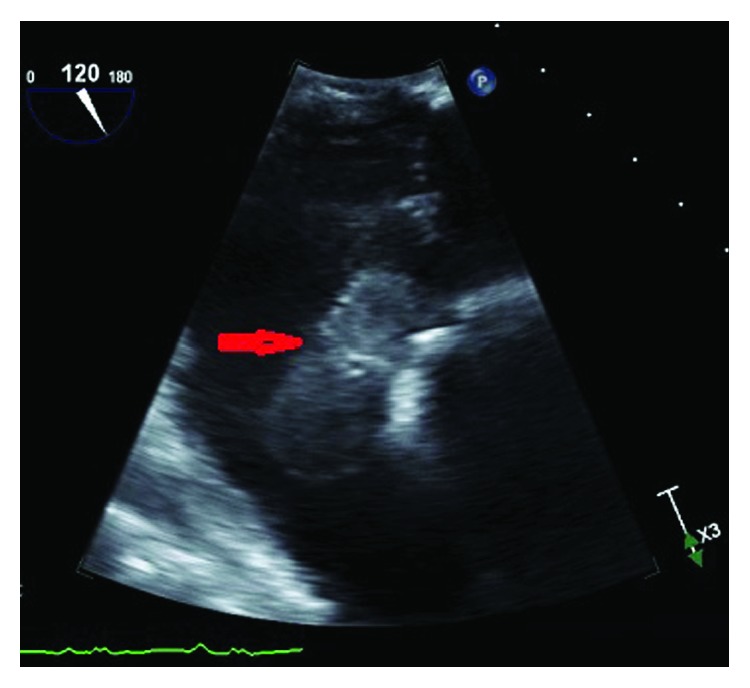
Focused midesophageal view of the RVOT on repeat TEE performed on hospital day 9. Note the large echodense structure measuring 3.4 cm × 1.6 cm that is independent of the tricuspid valve apparatus and is highly mobile with a narrow peduncle. The temporary pacemaker lead is also independent of this structure.

Our patient is a 64-year-old African American male with a past medical history significant for coronary artery disease status post percutaneous coronary intervention in the setting of a non-ST elevation myocardial infarction, hypertension, hyperlipidemia, diabetes mellitus type 2, chronic kidney disease stage III, and prior bariatric surgery. He presented to the emergency department with complaints of fatigue and progressive drainage from a left foot wound with associated pain. The patient was hypotensive (blood pressure 89/53 mmHg) and bradycardic (43 beats/minute) with complete heart block noted on telemetry monitoring and confirmed on 12-lead electrocardiogram. Initial laboratory tests were significant for leukocytosis (white blood cell count 32.1 K/mm^3^), anemia (hemoglobin 9.5 g/dl), and elevated BUN (51 mg/dl) and creatinine (3.6 mg/dl) from baseline. A temporary transvenous pacemaker was placed emergently in the cardiac catheterization laboratory. The patient was subsequently hospitalized for the management of septic shock, wet gangrene of the left second toe, and complete heart block. Empiric antibiotic therapy was initiated with vancomycin and piperacillin/tazobactam, and he underwent urgent toe amputation followed by left ankle disarticulation. Postoperatively, the patient developed respiratory failure requiring intubation. Initial blood cultures grew MRSA. Given persistent hemodynamic instability despite presumed infectious source control, echocardiography was performed to rule out infective endocarditis. Transthoracic echocardiogram (TTE) on hospital day 1 and TEE on day 3 were, however, noncontributory (Figure 1). Bacteremia was persistent through the second week of hospitalization on multiple sets of blood cultures, and antibiotic regimen was changed to ceftaroline. Due to the persistent bacteremia, a whole-body 18-florodeoxyglucose positron emission tomography/computed tomography (18F-FDGPET/CT) scan was then obtained on hospital day 8 which revealed multiple PET-avid lung nodules and cavitary lesions concerning for septic pulmonary emboli (SPE). TEE was then repeated on hospital day 9 revealing a highly mobile echodensity in the right ventricular outflow tract (RVOT) measuring 3.4 cm by 1.6 cm (Figure 2) without valve leaflet or chordae involvement. The patient was evaluated for surgical vegetectomy; however, his surgical risk was deemed prohibitive in the acute setting, and thus conservative management was pursued with antibiotic therapy alone. Infection control was not successfully achieved. From a Heart Team approach, the decision was made to attempt an AngioVac evacuation of the RV mass on hospital day 12.

**Figure 3:**
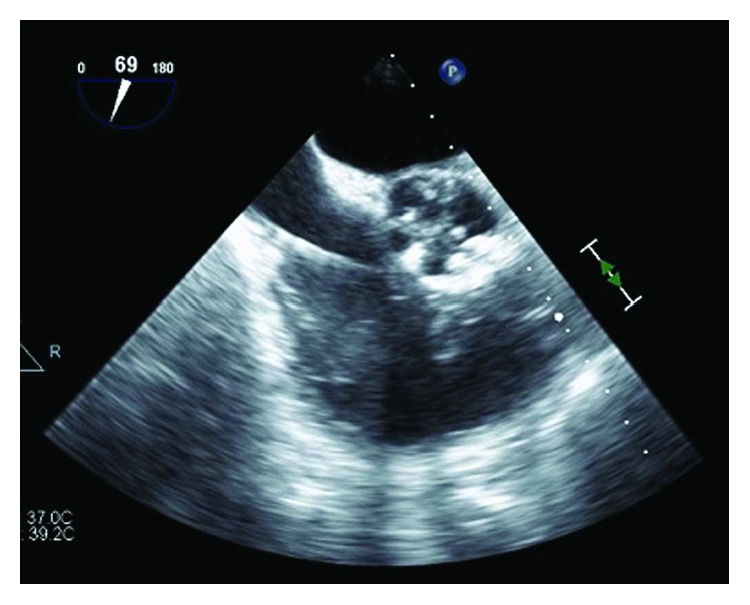
Midesophageal RVOT-focused view on intraoperative TEE immediately postaspiration using the AngioVac system (catheter can be visualized in this image.) Although residual vegetation is visible, there is approximately 90% resolution postaspiration.

Venous access was obtained at both femoral venous sites with 6-French sheaths, and a right internal jugular access was obtained for the replacement of a temporary pacemaker. The left 6-French sheath was changed out with a 17-French cannula after serial dilatation while the right sheath was replaced with a number 26 DrySeal Sheath. Using a Swan-Ganz catheter, a Swan wire was positioned into the right pulmonary artery (PA). Using an MPA catheter, a Lunderquist wire was positioned in the right PA. The AngioVac return cannula was then attached with a wet-to-wet seal to the perfusion pump. The return cannula was loaded over the Lunderquist wire after removing air from the cannula. Under TEE guidance, the return cannula was advanced into the right atrium. When the return cannula could not be advanced further into the RV outflow tract, it was pulled back and the Lunderquist wire was repositioned into the left PA using the MPA catheter. It was then possible to advance the aspiration catheter back and forth, and this resulted in the return of a large amount of material visually consistent with clots. Activated clotting time was maintained above 300 seconds throughout the procedure with heparin. Postprocedure TEE showed up to 90% resolution of the mass (Figure 3). Aspirated blood sampled from the PA was positive for MRSA. Antibiotic therapy was continued, and subsequent blood cultures were sterile. Resolution of complete heart block was verified on continued telemetry monitoring, and the temporary pacemaker was removed. The patient was eventually discharged in stable condition on hospital day 27.

## 3. Discussion

In the case presented here, persistent MRSA bacteremia and SPE on 18F-FDG PET/CT heightened the suspicion for endocardial infection. The RV outflow mass seen on repeat TEE appeared within 6 days of initial negative TEE and was suspected to be an infected thrombus or vegetation. The recommended initial management of right endocardial infection is antibiotics [4]. Surgical intervention is considered in specific situations. In our patient, persistent bacteremia for more than 7 days despite adequate antibiotic therapy was an indication for surgery. As is often the case in endocardial infections, our patient was too unstable clinically to undergo surgery. Recent reports have suggested that AngioVac aspiration could be a minimally invasive alternative to surgery and the decision was made to pursue this option.

The AngioVac venous drainage system, invented in 2012, was developed for the removal of fresh, soft thrombi or emboli from the vascular system. It was intended to be used as an alternative to anticoagulation, catheter-directed thrombolysis, mechanical thrombectomy, pharmacomechanical thrombectomy, or open surgical embolectomy [5, 6]. In a single-center analysis of 16 procedures, AngioVac aspiration achieved the removal of more than 70% of thrombi/masses with 67% and 100% success rate for the right atrial and iliocaval sites, respectively [7]. Similarly, Worku et al. analyzed case reports involving 56 procedures which showed the removal of 100% of material in 87% and 82% of iliocaval and intracardiac thrombus, respectively. On the other hand, the success rate for the removal of PE was remarkably low at 12.5% [8]. Pasha et al. had alternatively reported successful management of an acute massive PE in a hemodynamically unstable patient using the AngioVac system [9]. Other novel applications of the AngioVac catheter have been explored. Abubakar et al. published a detailed compilation of case reports including 65 procedures, noting an increasing popularity of AngioVac aspiration for vegetation debulking from right endocardial locations including the right atrium, tricuspid valve, pulmonic valve, and intracardiac device leads. In nearly all cases, successful vegetectomy led to infection control and clinical improvement [10]. In some cases, the AngioVac device has been used as a bridge to surgery [11, 12]. The most serious reported complication associated with the device is access site hematoma [8, 13].

With the finding of a large RV outflow mass initially suspected to be an infected thrombus or vegetation, we concluded that this was the source of persistent bacteremia and SPE. We decided that urgent thrombectomy or vegetectomy was the most viable option for infection control and for preventing further potentially catastrophic embolization into the pulmonary vasculature. Since our patient was at prohibitive perioperative risk, we elected to attempt retrieval by AngioVac aspiration. Following successful extraction of 90% of material visibly consistent with thrombus, we were able to achieve rapid control of the infection. To the best of our knowledge, this is the first report detailing successful AngioVac aspiration of a large infected thrombus from the RV outflow tract without serious complications.

## 4. Conclusion

When a right endocardial mass of any etiology is encountered in clinical practice, consideration should be given to the use of the AngioVac device if evacuation is desired. Use of this new device may be a safer alternative to surgical vegetectomy, particularly when the perioperative risk is prohibitive.
